# Experimental Investigation of the Dynamic Responses of Thin-Walled and Foam-Filled Steel Tubes Subjected to Repeated Impacts

**DOI:** 10.3390/ma17051018

**Published:** 2024-02-22

**Authors:** Jing Ge, Tingyi Luo, Jun Qiu

**Affiliations:** 1 School of Materials Science and Engineering, Tongji University, Shanghai 201804, China; gejing1212@tongji.edu.cn; 2 Jiangsu Hongyuan Science and Technology Engineering Co., Ltd., Changzhou 213161, China; 3 Guangxi Beitou Highway Construction and Investment Group Co., Ltd., Nanning 530028, China; luoty0116@163.com; 4 Key Laboratory of Advanced Civil Engineering Materials (Tongji University), Ministry of Education, Shanghai 201804, China

**Keywords:** dynamic response, horizontal impact test, thin-walled tube, foam-filled tube, repeated impacts, PU foam

## Abstract

In this study, a horizontal impact setup was used to measure the dynamic responses of specimens fixed on a reaction wall and subjected to repeated impacts generated by a large-tonnage impactor. The contact force, deformation process, energy absorption, and other properties of two specimens (a thin-walled steel tube and foam-filled steel tube) were thoroughly investigated. The results demonstrated that the thin-walled tube’s properties were consistent with the four-phase and six-phase deformation models and that the foam-filled tube’s properties were consistent with the two-phase deformation model. In the early stages of the experiment, the foam-filled and thin-walled tubes were similar in terms of the contact force and energy absorption. However, when the polyurethane (PU) strain reached 0.8, the PU significantly increased the support of the tubes, reduced the contact force (by extending the contact time), and increased the energy absorption capacity by 33.6–43.5%. The crush curves of the specimens were in agreement for cases involving multiple impacts, as well as for one impact with the same impact of kinetic energy. The crush curves can be used to assess the actual performance of crashworthy devices. Furthermore, after repeated impacts, the foam-filled tube exhibited a pseudo-shakedown behavior.

## 1. Introduction

The navigation challenges between ships and bridges in the water constitute a natural contradiction. Ship impacts threaten the structural stability of bridges, and transient impacts have significant potential to cause structural damage to bridges [1]. Several studies have investigated the installation of crashworthy devices around bridge piers [2,3,4,5], which are intended to absorb energy and increase the structural safety of bridges.

Thin-walled (TW) tubular components are considered the best energy-absorbing structures for lateral loading. The compression and collapse models of thin-walled tubes have been thoroughly evaluated. For example, DeRuntz et al. [6] assumed that the wall of the tube is rigid and does not deform except at the hinge, and the deformation and collapse of a circular ring involves four plastic hinges. The six-phase deformation model with four phases in the flattened contact zone and two phases in the parallel mid-plane sections was proposed by Oliveira J G et al. [7]. Reid and Reddy [8] enhanced the DeRuntz et al. [6] models by accounting for the strain hardening effect. These two models were confirmed by Xu et al. [9] via tests, which demonstrated that a circular tube deforms according to a six-phase deformation model at both high and low velocities. In order to address the issue of deformation analysis for tubular elements under pinching loads, Fonseca et al. [10] proposed an alternative formulation based on a multi-nodal finite tubular ring element, which has eight degrees of freedom overall for each section under consideration.

**Figure 1:**
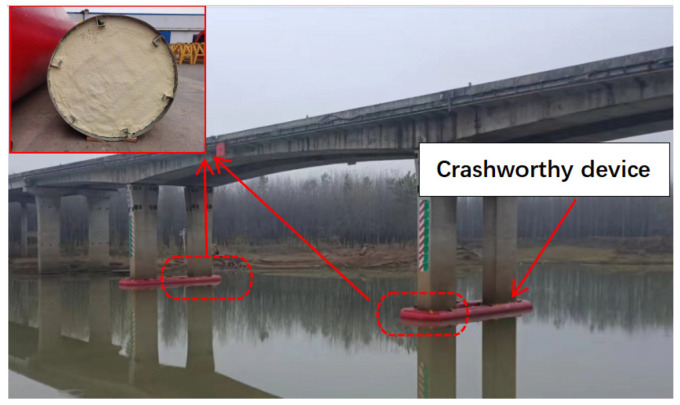
Crashworthy device of bridge.

Crashworthy devices should have a narrow maximum size to reduce the volume of the navigable water they occupy. One key strategy for improving the energy absorption is to fill the interior of the tube with materials such as aluminum foam and polyurethane (PU) [11,12] (as shown in Figure 1). Rogala et al. [13] investigated the mechanical properties of aluminum porous structure using static axial compression tests and found that aluminum foam is a better energy absorber. Although these materials are prone to significant deformation under loads, they offer a strong resistance to thin-walled shell deformation once they fill the structure, substantially increasing both its crashworthiness and energy absorption capacity. An analytical technique based on the stress and deformation of laminated composite circular tubes subjected to transverse loading was presented by Xia et al. [14]. That study used two analytical techniques based on multilayer stacking theory and curved composite beams to theoretically prove that the composite circular tube can increase the energy absorption capacity. Fan et al. [12] conducted dynamic transverse crush tests on sandwich tubes that were filled with aluminum foam. The findings demonstrated that, compared to the quasi-static process, the energy absorption of the sandwich tube subjected to dynamic compression was significantly higher when the impact velocity was greater than a critical value. The lateral compression collapse properties of a nested foam-filled tube system with a unique cross section were examined by Niknejad et al. [15]. The inner circular tube was made of brass and aluminum alloys, the notched portion was made of an aluminum alloy, and the filler was made of PU foam. The study showed that filling tubes with foam is a useful technique for improving the energy absorption behavior of tubes subjected to transverse loads. Bilston et al. [16] investigated the energy absorption characteristics of circular tubes with Terocore^®^ structural epoxy foam and different aluminum alloys through dynamic three-point bending testing. The findings showed that the lightweight foam-filled material reduces the folding/bending tendency of the wall strength/thickness combination and stabilizes the deformation of thin-walled beams, thereby boosting the beams’ specific energy absorption and load-carrying capability. When PU foam was poured into E-glass/vinyl ester composite tubes, Niknejad et al. [17] discovered that the filling significantly increased the composite tubes’ energy absorption capacity. Comparing the composite tubes filled with PU foam to their empty counterparts, the energy absorption capacity of the filled tubes increased from 338% to 957%. Specifically, the increase in the energy absorption capacity was caused by the adhesion of the PU to the tube wall. Rogala et al. [18] studied the use of aluminum–silicon carbide composite foam as a filler for thin-walled aluminum profiles, and the effect of foam length on energy absorption behavior was investigated by means of experiments and finite element simulations.

Although crashworthy devices are frequently subjected to repeated impacts by small ships traveling at low velocities, they can also be repeatedly struck by drifting objects and ice jams. These repeated impact loads have a number of traits in common, including the induction and accumulation of plastic deformation in the structure (which may not result in an instantaneous structural failure). Zhu [19] conducted repeated impacts on aluminum foam sandwich panels. The behavior of sandwich panels made of aluminum foam and subjected to single and repeated repetitive drop hammer blows was examined. Kim et al. [20] investigated the effects of repetitive low-energy impact on mechanical behavior of glass fiber-reinforced PUF. In addition, Ghajar et al. [21] performed experimental and numerical tests on rectangular aluminum plates. They observed the plate deformation process from plastic deformation to penetration and detected an increase in the stiffness. They also performed analyses and comparisons on the changes in the contact force and displacement for various impact loads. Li et al. [22] carried out an experimental and numerical study on the life prediction and cracking behavior of simply supported 18Cr2Ni4WA steel beams subjected to repeated impact loads.

Jones [23] proposed the pseudo-shakedown phenomenon for rigid, perfectly plastic rectangular plates subjected to repeated impacts. Shen et al. [24] examined the number of repeated dynamic loads necessary to reach the pseudo-static condition and illustrated the pseudo-shakedown phenomenon for rigid, perfectly plastic beams and plates subjected to repeated dynamic stresses. Using rigid-plastic analysis, Jones and Norman [25] expanded the research on the pseudo-shakedown phenomenon to include circular and rectangular plates subjected to repeated impacts of the same mass. However, not all repeated impacts result in a pseudo-shakedown. For instance, several repeated impact studies [19,20,21,22] did not report the pseudo-shakedown phenomenon. As a result, the peak contact force, maximum displacement, impactor rebound velocity, and contact time of the structure subjected to repeated impacts essentially remains constant, according to He and Soares’ definition of pseudo-static phenomena [26].

Bridge crashworthy devices are often designed based on impact energy that represent ships at higher speeds, but they are often subjected to multiple low-speed impacts over their lifetimes. These repeated impact loads have some common characteristics, they may not lead to immediate structural failure, as they are usually minor impacts, but these smaller-scale impacts may cause and accumulate plastic deformation in the structure. Therefore, the protective performance of crashworthy devices and their own structural safety based on cumulative damage scenarios is an important issue worth investigating. Once the plastic deformation accumulates to a certain level, the crashworthy device’s structure will eventually fail. The effect of damage from repeated impacts on the energy dissipation effect of the crash protection device, and the damage assessment of the accumulated damage to the safety of the structure itself, is an important issue. When repeated impacts occur, the inelastic deformation from the previous impacts and the resulting residual stresses have an impact on the behavior of the following impacts. In addition, material properties, boundary conditions, etc. may change. Therefore, the dynamic response of a structure under repeated impacts involves many complexities that necessitate this study.

In this paper, the dynamic responses of thin-walled and foam-filled steel tubes subjected to repeated impacts were investigated experimentally. The experimental results were analyzed and discussed with the aim of elucidating the response mechanism and providing useful information for understanding the behavior of the structure in response to repeated impacts. The rest of this paper is organized as follows. Section 2 describes the experimental method, Section 3 presents the experimental results, Section 4 contains the conclusions.

## 2. Experimental Method

### 2.1. Test Specimens

**Figure 2:**
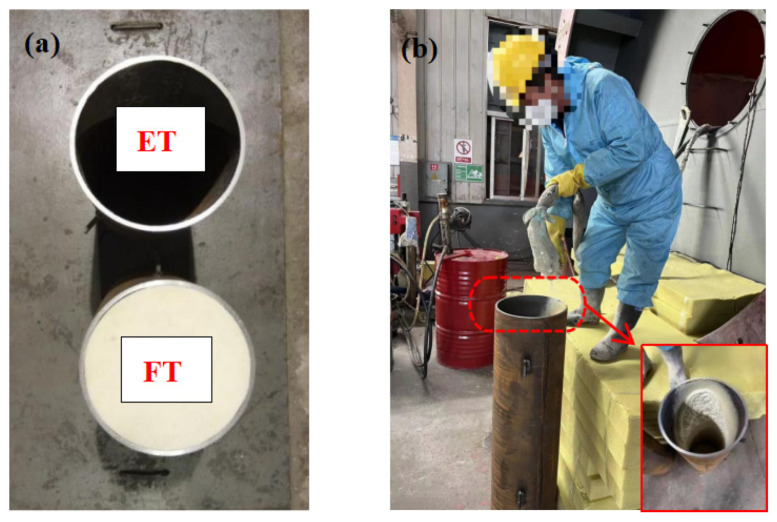
Test specimens: (**a**) ET and FT specimens and (**b**) PU spray molding.

Four test specimens were prepared for the impact loading tests. The specimens were tubes measuring 1000 mm in length and 300 mm in diameter. The specimens labeled ET-1 and ET-2 were made of 20# steel tubes with thin walls that were 8 mm thick, and the ratio of diameter to thickness (D/t) is 37.5. The FT-1 and FT-1 specimens consisted of the same tubes, but they were filled with PU foam instead (as shown in Figure 2a). The PU was created via spray molding, a procedure that involves premixing polyester polyolare and isocyanate with high-pressure air, which was followed by spraying the mixture into the interior of a steel pipe. The ratio of isocyanate to polyester polyolar was 1.05:1, as indicated in Figure 2b.

**Figure 3:**
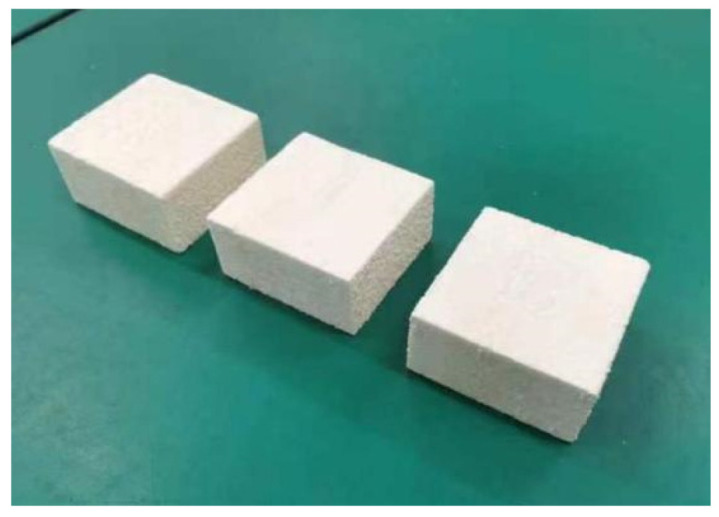
PU specimens.

**Figure 4:**
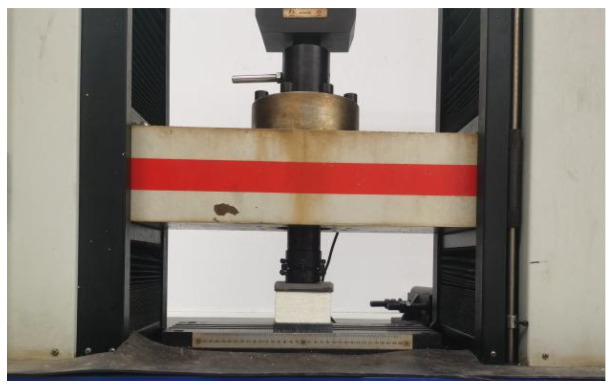
Compression test.

**Figure 5:**
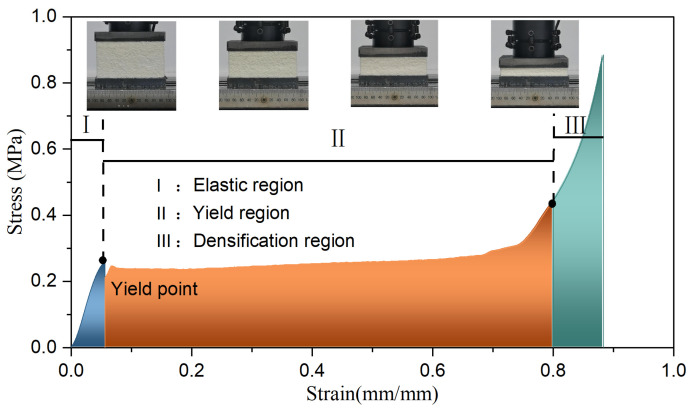
Compressive stress–strain curve of the PU foam.

Following the Chinese code [27], the PU foam was made into a specimen of 100 mm × 100 mm × 50 mm (Figure 3), and an axial compression test was performed using a DNS-100KN-type electronic universal testing machine (Tongji University, Shanghai, China) (Figure 4) with a loading speed of 10 mm/min. The compressive stress–strain curve of the PU is shown in Figure 5. The compression process of the PU foam was divided into three stages: (I) the initial linear elastic response region, (II) the platform collapse with large deformation region, and (III) the densification region. First, the pore structure was deformed and exhibited stress concentration. As the load increased, the pore wall was bent, and the elastomer molecular chain broke after being subjected to stress, resulting in cracks on the pore wall and the collapse of the pore structure, which then extended along the laminar pattern. Finally, the entire specimen was compacted. Stage deformation occurred in a very small area, and its strain was less than 0.055. The compressive stress–strain curve for this stage could be approximated as a straight line, and the PU foam behaved elastically after being subjected to external forces. As the deformation increased, the deformation zone developed and collapsed layer by layer, which was the result of the joint action of the bending and folding of the hole wall. The change in the stress was relatively smooth at this stage, and the stress reached 0.45 MPa when the strain reached 0.8. When the walls of the specimen pores were squeezed together, the specimen entered the densification region because the substrate that constituted the wall of the pore was compressed, resulting in a sharp increase in stress.

### 2.2. Test Setup

**Figure 6:**
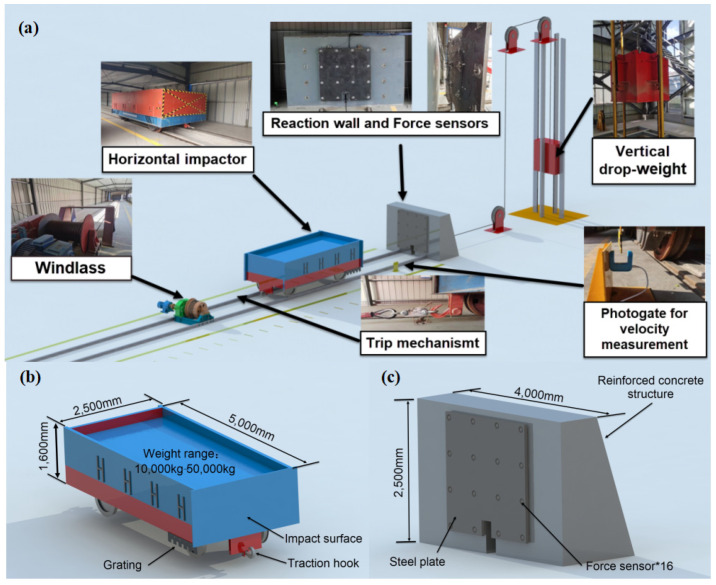
Experimental setup: (**a**) horizontal impact system, (**b**) horizontal impactor, and (**c**) reaction wall.

**Figure 7:**
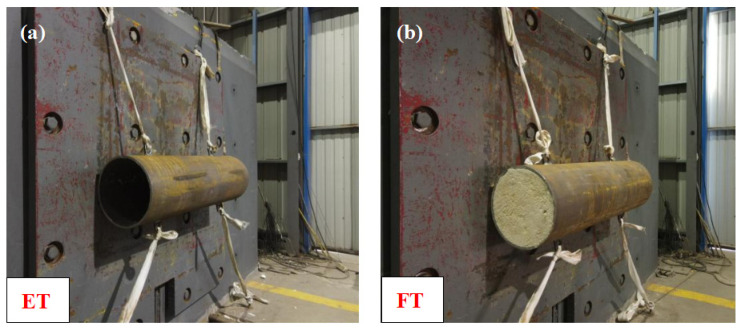
Experimental setup: (**a**) ET specimen and (**b**) FT specimen.

Impact tests were carried out using the horizontal impact system (Figure 6) of the Jiangsu Province Bridge Collision Prevention Engineering Technology Research Center in China. The test system consisted of a vertical drop-weight device combined with a horizontal impactor traction device (Figure 6a). A windlass was used to pull the horizontal impactor via the steel cable (Figure 6b), and the horizontal impactor synchronously pulled the vertical drop weight through the fixed pulley set. During the test, a vertical drop weight was used to pull the horizontal impactor against the specimen while it was fixed to the reaction wall (Figure 7) and the trip mechanism broke the link between the windlass and the horizontal impactor. Simultaneously, piezoelectric sensors were arranged on the reaction wall (Figure 6c) to measure the impact of the specimen on the reaction wall.

The horizontal impactor was made of a steel plate, steel channel, and I-beam welded together. The impact velocity (and thus the kinetic energy) of the horizontal impactor could be adjusted by controlling the height of the vertical drop weight and adjusting the counterweight of the horizontal impactor. A total of 16 force sensors were used in the experiment, and the range of each force sensor was 0–600 KN. The sampling frequency of the data collector was 100 KHZ.

### 2.3. Impact Scenarios

Table 1 shows the impact mass (m_s_), initial impact velocity (v_s_), and kinetic energy (E_o_) measured for each specimen. For each specimen, the impact tests were repeated until it was completely deformed. For specimens made of identical materials (i.e., ET-1 and ET-2, FT-1 and FT-2), the impact mass was held constant while the impact kinetic energy of the first specimen was twice that of the second specimen. This was performed in order to study the influence of the impact velocity on the properties of repeated impacts. After several tests, the coefficient of kinetic friction between the impactor and track decreased, which may have caused slight variations between the impact velocities in the actual tests and the nominal impact velocities.

## 3. Analysis of Results

### 3.1. Summary of Results

**Figure 8:**
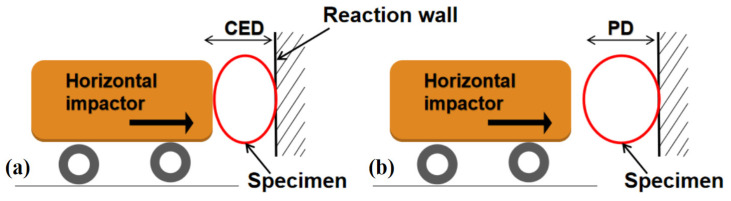
Deformation of specimen: (**a**) MD and (**b**) PD.

An overview of the impact mass, impact velocity, maximum deformation (MD), and permanent deformation (PD) results is presented in Table 2, Table 3, Table 4 and Table 5. The contact force–time curve was used to calculate the maximum deformation (MD), and manual measurements were used to determine the permanent deformation (PD). These deformations are illustrated in Figure 8. The kinetic energy *E_o_* of the initial impact, which is expressed as

(1) $$E_{o}={(m}_{s}\times V_{s}^{2})/2,$$

was determined from the initial velocity and mass of the impactor. In addition, the kinetic energy absorbed *E_d_* by the specimen was obtained by integrating the contact force–deformation curve according to

(2) $$E_{d}=\int_{0}^{l}Fdl$$

The test setup itself also absorbed a small amount of the energy, but this fact was ignored in this study.

### 3.2. Dynamic Deformation Mechanism

**Figure 9:**
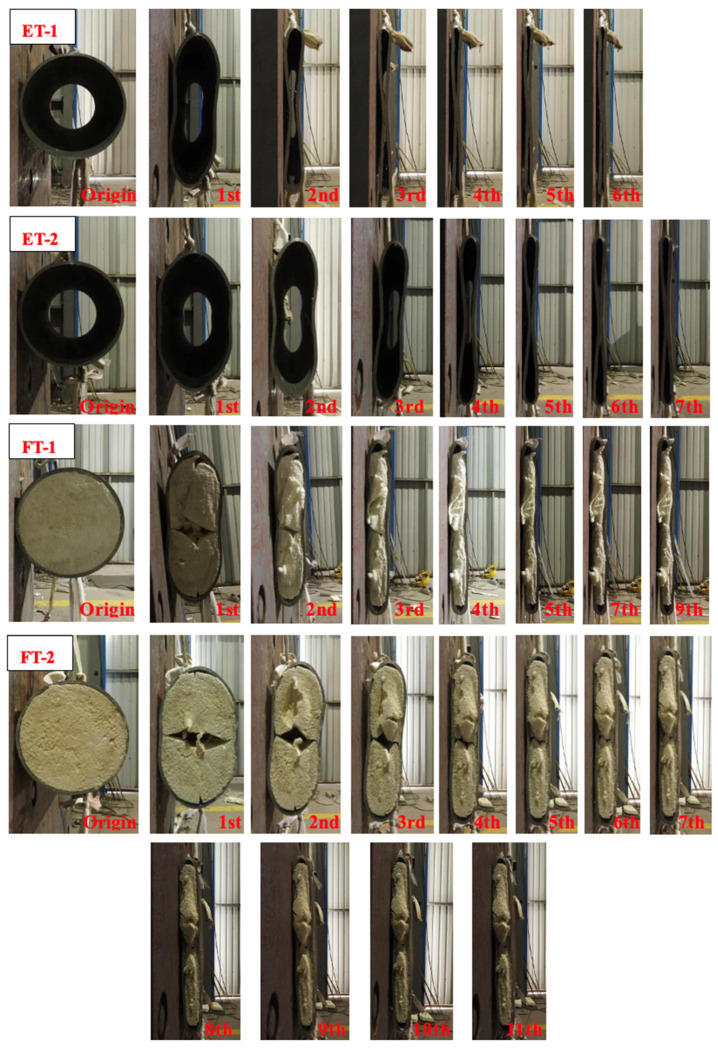
Deformation of the four specimens after repeated impact tests.

**Figure 10:**
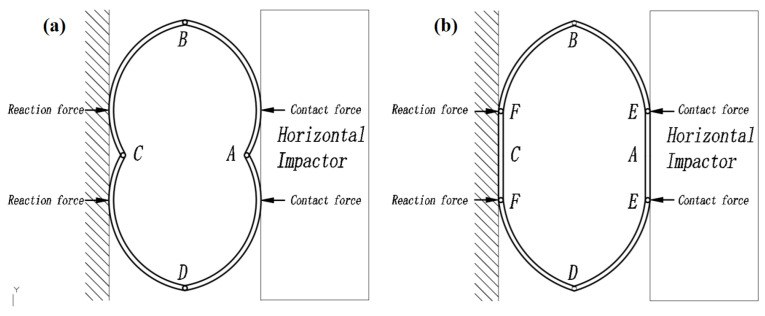
Compression and collapse models of tubes subjected to lateral loading: (**a**) the four-phase deformation model and (**b**) the six-phase deformation model.

Figure 9 shows the deformation of the four specimens after being subjected to repeated impact tests. The properties of specimens ET-1, ET-2, FT-1, and FT-2 reflected different deformation models (Figure 10). At an impact velocity of 1.5 m/s, the cross-sectional shape of specimen ET-2 was deformed from a circle to an ellipse after the first impact, and no concave portion was observed on the collision side or the opposite side. Points B and D remained almost stationary with respect to the center of the ring, which is a typical characteristic of the six-phase deformation model (Figure 10b). As the number of impacts increased, significant depressions appeared at points A and C, and distinct “V” blocks appeared on both the impact side and the opposite side. At least four plastic hinges (points A, B, C, and D) were identified (Figure 10a), which is consistent with the four-phase deformation model proposed by DeRuntz et al. [6] and the dynamic deformation model proposed by Owens and Symonds [28]. After the fourth impact, the “V” blocks at points A and C still existed, but points A and C were reunited. Points A and C were no longer considered plastic hinges, and the curvature at points B and D increased. The fifth, sixth, and seventh impacts, at which time the deformation of the tube no longer reflected the four-phase deformation model proposed by DeRuntz et al. [6], the circular arcs AB, BC, CD, and DA were deflated by the horizontal impactor, and were gradually deformed from circular arcs to straight lines. When the impact velocity was 2.13 m/s, the deformation model of the first impact of specimen ET-1 was essentially the same as that of the two impacts of specimen ET-2 (reflecting a four-phase deformation model), and the deformation trend was the same as that of specimen ET-2 as the number of impacts increased. Xu et al. [9] suggest that the manifestation of the six-phase deformation model or the four-phase deformation model is primarily determined by the impact velocity; the six-phase model manifests when the impact velocity is lower than 40 m/s, and the concave four-phase deformation model occurs for velocities higher than 60 m/s. However, the six-phase model should manifest before the four-phase model. When the impact kinetic energy was insufficient, the specimen was deformed from a circle to an ellipse, and there was no concavity on the collision side or the opposite side. However, when the impact kinetic energy exceeded the threshold value, points A and C exhibited internal concavity, which manifested as a four-phase deformation model. Therefore, the energy was the main factor controlling the appearance of the two deformation models. These results are consistent with those of Xu et al. [9], which indicated that the deformation at low speed (low energy) was characterized by the six-phase model and the deformation at high speed (high energy) was characterized by the four-phase model.

The deformation models of the FT specimens differed significantly from those of the ET specimens. For the first impact, which had a velocity of 1.5 m/s, the deformation of specimen FT-2 was basically the same as that of specimen ET-2, which exhibited a six-phase deformation model. In contrast, the PU in the center of the specimen exhibited significant cracking, and the curvature of points B and D became larger. For the second and third impacts, slight internal concavity was observed at points A and C; however, the “V” block was significantly smaller than that of the ET-2 model. In addition, the curvatures of points B and D were significantly smaller than those in the ET model because of the support provided by the PU. Starting from the fourth impact, the “V” blocks at points A and C were almost invisible, which was significantly different from the deformation of the ET-2 model. Specimen FT-2 exhibited a two-phase deformation model, in which the curvature of the articulation points at points B and D was significantly larger. For the fifth to eleventh impacts, the basic deformation mechanism of the specimen did not change significantly, and the difference was that the spacing between points A and C was continuously compressed, while at the same time the curvature of points B and D continued to increase. For the eleventh impact, the contact force and permanent deformation of the specimen changed minimally, initiating a pseudo-shakedown state. In this test, the deformation model of the tube was constrained by filling the tube with PU material, and the filling of the PU inhibited the depression at points A and C, which allowed the tube to conform to the two-phase model at a very low velocity. The impact kinetic energy of specimen FT-1 was twice as much as that of specimen FT-2, and the corresponding deformation was larger than that of FT-2. The difference mainly occurred at the points at which the PU became detached (points B and C.) The PU at points B and D in the FT-1 model was more deflated than that in the FT-2 model for the same impact kinetic energy, which indicated that the strain rate associated with a higher impact velocity affected the properties of the PU. Fan et al. [12] also found that tubes filled with aluminum foam were less prone to concavity at points A and C after impact, which suggests that concavity can be avoided as long as the interior of the tubes is filled with either aluminum foam or PU.

### 3.3. Deformation vs. Impact Number

**Figure 11:**
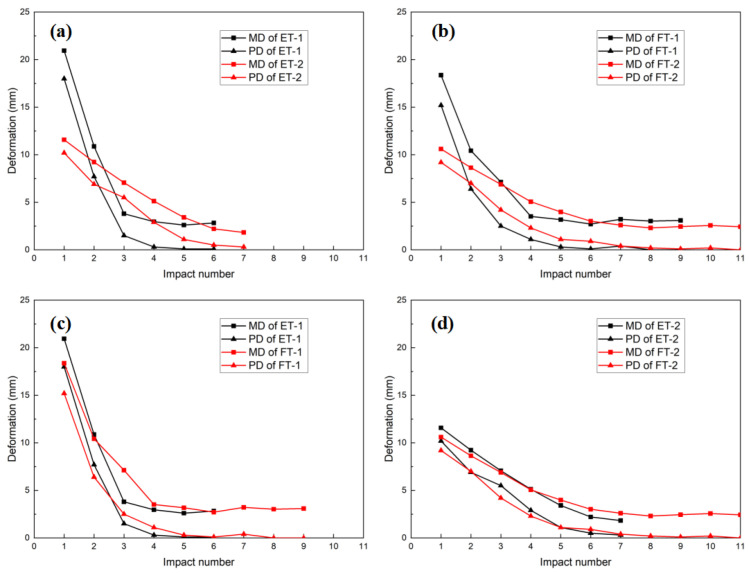
Comparison of deformations: (**a**) ET-1 vs. ET-2, (**b**) FT-1 vs. FT-2, (**c**) ET-1 vs. FT-1, and (**d**) ET-2 vs. FT-2.

The maximum deformation (MD) and permanent deformation (PD) of the ET and FT specimens as a function of the number of impacts are shown in Figure 11a,b. The maximum PD of specimen ET-1 was 27.7 mm after the sixth impact, and the maximum PD of specimen ET-2 was 27.4 mm under the seventh impact. After the ninth impact, specimen FT-1 reached a maximum PD of 26 mm, and after the eleventh impact, specimen FT-2 reached a maximum PD of 25.7 mm.

The largest deformations occurred for both the FT and ET specimens immediately after the first impact. The MD and PD of specimen ET-1 were 20.9 mm and 18 mm, respectively. The MD and PD of specimen ET-2 were 11.5 mm and 10.2 mm, respectively. The MD and PD of specimen FT-1 were 18.4 mm and 15.2 mm, respectively. The MD and PD of specimen FT-2 were 10.6 mm and 9.2 mm, respectively. Because of the PU filling, the MD decreased by 13.6% and the PD decreased by 18.4% at a velocity of 2.13 m/s after the first impact. At a velocity of 1.5 m/s, the MD was reduced by 8.5% and the PD was reduced by 10.8%. This indicates that the PU-filled tubes acted as supporting elements that reduced the deformation during impact; however, the amount of support offered by the PU diminished as the impact velocity decreased.

Specimen ET-1 and ET-2 were completely crushed after four or five impacts; however, its MD and PD were larger than those of specimens ET-1 and ET-2 after the third impact. This occurred because specimens FT-1 and FT-2 reached a pseudo-shakedown state after multiple impacts, the PU was compressed into the densification stage, and the elasticity modulus increased quickly, which significantly decreased the deformation of the tubes (Figure 11c,d). This is consistent with the pseudo-shakedown state achieved for steel and aluminum alloy beams using drop hammer specimens, as observed by Xu and Soares [26]. These results are important for assessing the residual performance of crashworthy devices after repeated impacts.

After the second impact on specimen ET-2, its total input energy was nearly identical to that of specimen ET-1 after its first impact. The cumulative MD of specimen ET-2 was 20.7 mm, and its cumulative PD was 17.1 mm, whereas the MD of specimen ET-1 was 20.9 mm, and its PD was 18 mm with the same input energy. Evidently, after a single impact, the steel structure essentially deformed in the same manner as it did after a series of strikes with the same impact kinetic energy. Nevertheless, because steel is sensitive to velocity, the deformation of a low-velocity impact was less severe than that of a high-velocity impact with the same energy.

After the second impact on specimen FT-2, its total input energy was approximately equal to that of specimen FT-1 after its first impact. Specimen FT-1 had an MD of 18.3 mm and a PD of 15.2 mm after its first impact, whereas specimen FT-2 had a cumulative MD of 19.2 mm and a cumulative PD of 16.2 mm following the second impact. By comparison, the cumulative PD of specimen FT-2 after its fourth impact was 22.7 mm, that of specimen FT-1 after its second impact was 21.6 mm, that of specimen FT-2 after its sixth impact was 24.7 mm, and that of specimen FT-1 after its third impact was 24.1 mm. Consistent with the pattern exhibited by specimens ET-1 and ET-2, specimens FT-1 and ET-2 exhibited an almost identical deformation when subjected to the same input energy. However, these results showed that the deformation resulting from multiple low-speed impacts with the same energy input was greater than the deformation of a few high-velocity impacts with the same energy input. According to this analysis, at higher velocity (2.13 m/s), the detachment of the PU from the steel tube at points B and D is significantly greater than at lower velocities. This observation is consistent with the test described in Section 3.2, which also indicated that the separation of the PU from the tube at points B and D was more significant at higher speeds (2.13 m/s) than at lower speeds.

### 3.4. Contact Force–Time Measurements

**Figure 12:**
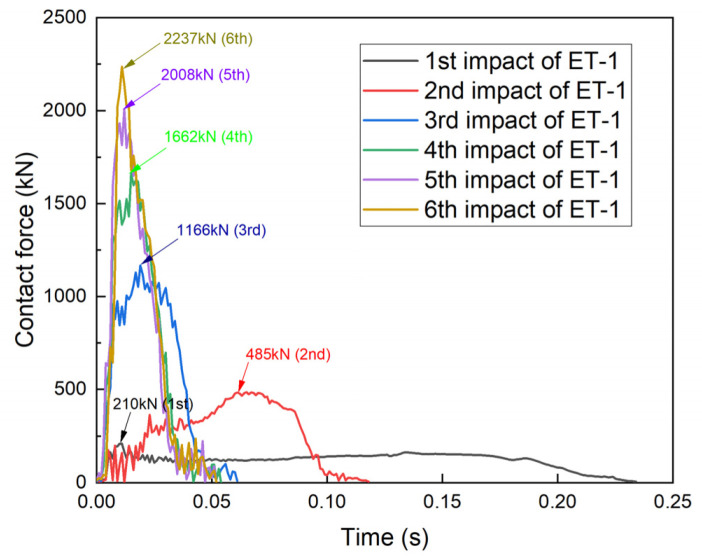
Contact force–time curve for specimen ET-1.

**Figure 13:**
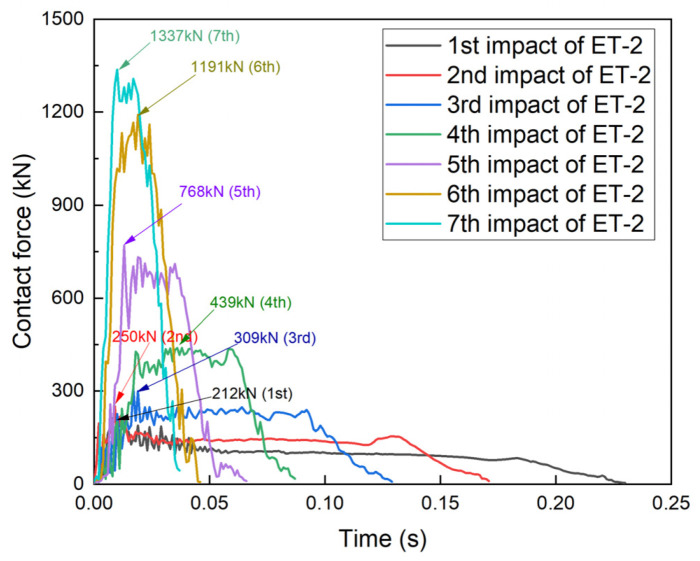
Contact force–time curve for specimen ET-2.

**Figure 14:**
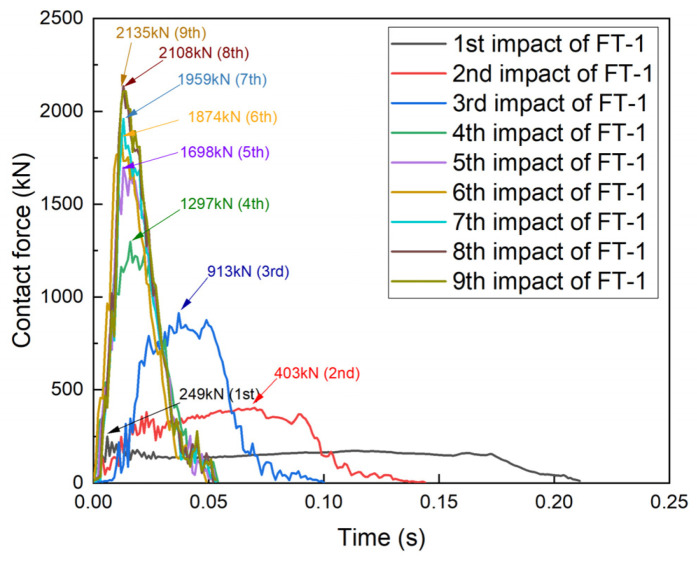
Contact force–time curve for specimen FT-1.

**Figure 15:**
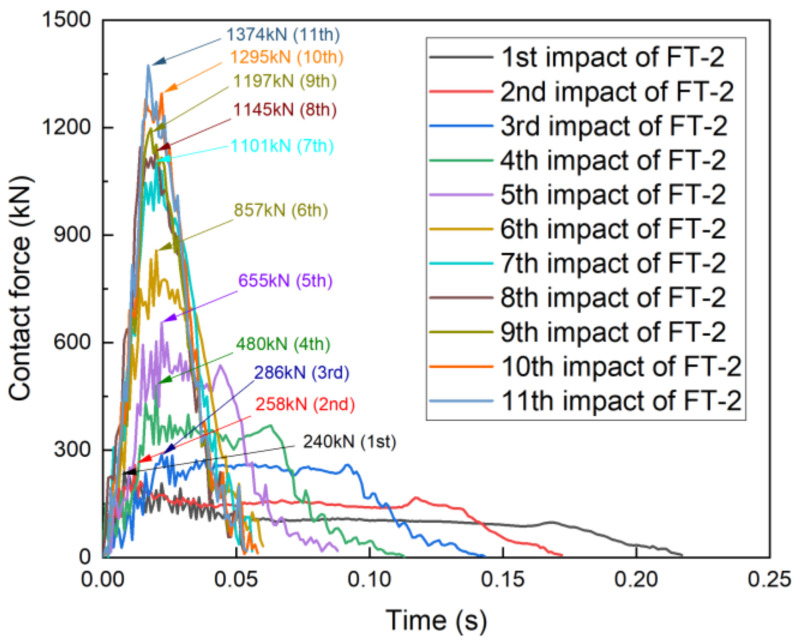
Contact force–time curve for specimen FT-2.

The contact force–time curves of the four specimens are shown in Figure 12, Figure 13, Figure 14 and Figure 15, and all four specimens exhibited a pattern of increasing contact force with decreasing contact time after repeated impacts. The contact force–time curve of specimen ET-1 after repeated impacts is shown in Figure 12. The impact time of 0.23 s and the average contact force of 200 kN after the first impact show that the specimen exhibited favorable plastic deformation. For the second impact, the peak of the contact force (485 kN) appeared in the second half of the impact duration (0.065 s), and the curvature at points B and D became larger. As the number of impacts increased, the contact area between the specimen and the counterforce wall increased, and the oscillations of the contact force subsided. The peak forces of the fourth, fifth, and sixth impacts increased from 1662 kN and 2008 kN to 2237 kN, and the total impact duration was about 0.04 s in all cases. However, the time at which the peak contact force occurred became earlier as the number of impacts increased.

The contact force–time curve of specimen ET-2 was similar to that of specimen ET-1 (Figure 13). The impact durations of both ET-1 and ET-2 were 0.04 s for the same crushed severity, but the peak contact force of specimen ET-2 was 1337 kN, which was much lower than that of specimen ET-1. This indicates that the peak contact force depends on the velocity and energy of the impactor when the thin-walled tube is crushed.

**Figure 16:**
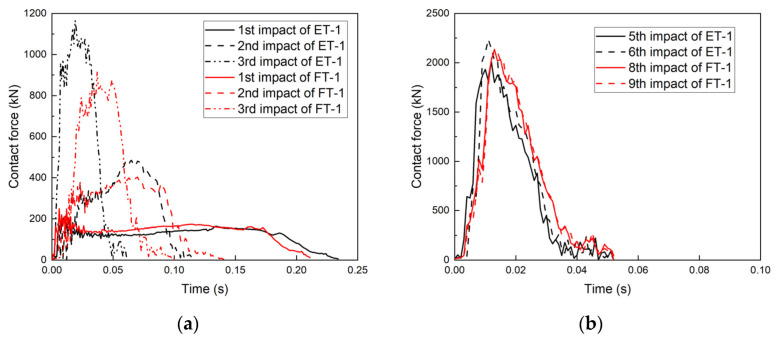
Contact force–time curves for specimens ET-1 and FT-1: (**a**) first to third impacts and (**b**) fifth to ninth impacts.

A comparison of specimens FT-1 and ET-1 (Figure 16) revealed that the impact duration of specimen FT-1 at the first impact was 0.21 s, which was smaller than that of specimen ET-1 (0.23 s), and the average value of the contact force was 200 kN. The peak contact force decreased from 485 kN to 403 kN, and the impact duration increased from 0.12 s to 0.14 s for the second impact. For the fourth impact, the difference between FT-1 and ET-1 became more obvious; the peak contact force of specimen FT-1 decreased from 1166 kN to 913 kN and the duration of the impact increased from 0.06 s to 0.1 s. At the beginning of the second impact, the peak contact forces of specimen FT-1 were lower than those of specimen ET-1, and the impact durations of FT-1 were longer than those of ET-1. This occurred primarily because of the PU filling, which prevented the transformation of the specimen from the six-phase deformation model to the four-phase deformation model. Points A and C were not in direct contact, and specimen FT belonged to the elastic-plastic region, thus demonstrating superior cushioning performance.

**Figure 17:**
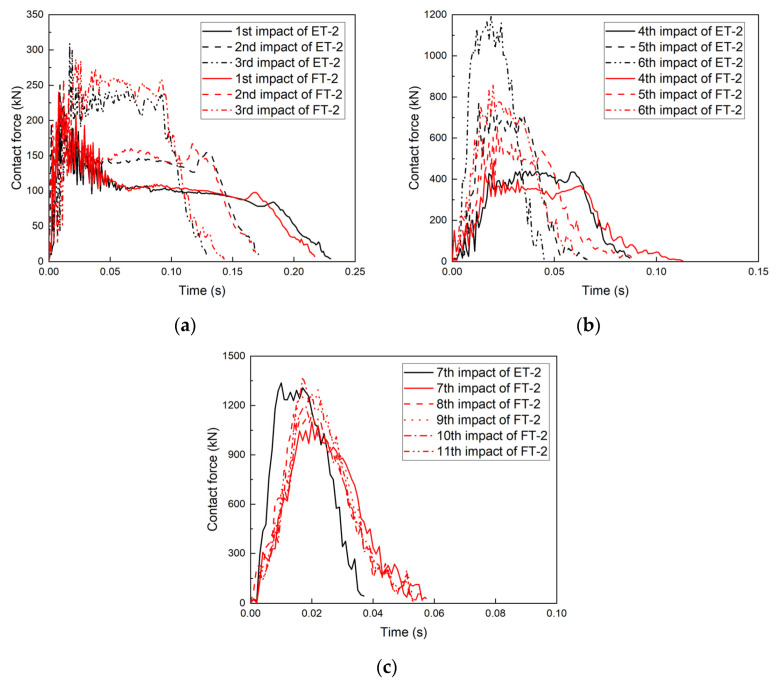
Contact force–time curves for specimens ET-2 and FT-2: (**a**) first to third impacts, (**b**) fourth to sixth impacts, and (**c**) seventh to eleventh impacts.

Comparing specimens FT-2 and ET-2 from the first to third impacts, the trends of the peak contact force and contact force duration were essentially the same, with FT-2’s contact force being marginally greater than that of ET-2 (Figure 17). The contact force of specimen FT-2 was significantly lower than that of specimen ET-2 from the fourth impact, and the contact force duration of specimen FT-2 increased from 0.086 s to 0.11 s. At the beginning of the fifth impact, the contact force of specimen FT-2 was significantly lower than that of specimen ET-2, and the contact force duration of FT-2 was significantly longer than that of ET-2, which was consistent with the trends for specimens FT-1 and ET-1. The peak contact force of specimen ET-2 during the sixth impact was 1196 kN, as compared to only 860 kN for specimen FT-2.

These results indicated that at different velocities, both PU-filled specimens exhibited superior cushioning properties compared to the unfilled specimens because of their ability to lower the peak contact force. The magnitude of the seventh to eleventh impacts for FT-2 remained almost the same, indicating that the specimen reached the pseudo-shakedown state. The peak contact forces after repeated impacts on FT-1 and FT-2 were 2135 kN and 1374 kN, respectively. Even for the PU-filled tubes, the peak contact force was primarily affected by the velocity and energy of the impactor in the case of complete collapse, which is consistent with the trends observed for thin-walled tubes.

### 3.5. Contact Force vs. Deformation

**Figure 18:**
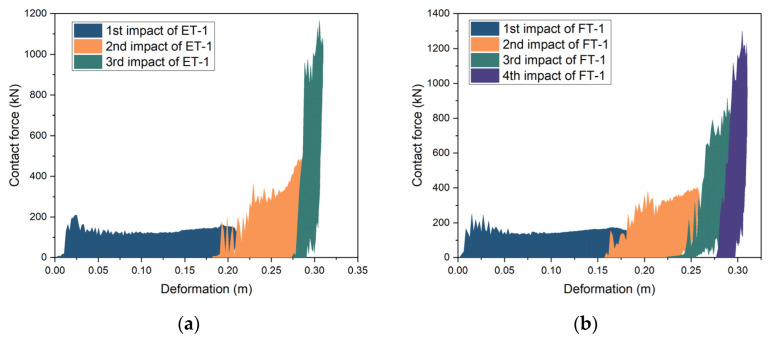
Connecting contanct force–deformation curves: (**a**) specimen ET-1 and (**b**) specimen FT-1.

**Figure 19:**
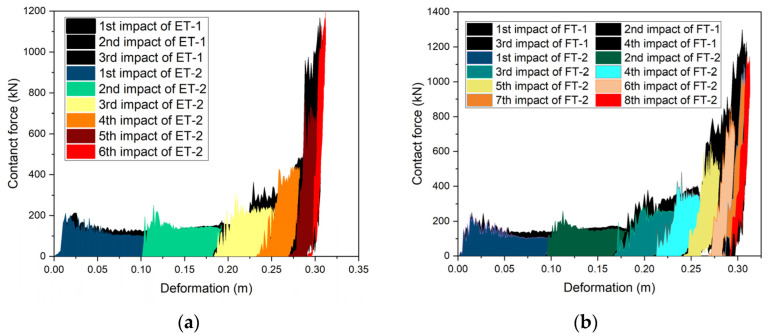
Comparison between connecting contact force–deformation curves: (**a**) specimens ET-1 and ET-2 and (**b**) specimens ET-2 and FT-2.

All four specimens exhibited progressive plastic crushing owing to repeated impacts, which did not change the crush stiffness of the specimens. To characterize and visualize the process of repeated impacts, the specimen’s crushing performance was determined by connecting the contact force–deformation curves from the repeated impact tests. The increases in the deformation resulting from each impact were added to a cumulative deformation, which was then synchronized with the corresponding contact force data. Removing the unloading and reloading portions of the curve resulted in a complete crush curve for each specimen (Figure 18a,b). The contact force–deformation curves for the six impacts on specimen ET-2 were connected and compared with the contact force–deformation curves for the three impacts on specimen ET-1. The two sets of curves were in good agreement (Figure 19a), in accordance with the rule obtained by Kantrales [29] after several impacts on the bow of a ship using a pendulum hammer.

If the input energy is limited and the crush curve of the specimen cannot be obtained by a single impact, several repeated impacts can be executed to connect the force–deformation curves, allowing the crush curve of the specimen to be produced. This is of great significance for evaluating the performance of crashworthy devices via direct tests. A comparison between the force–deformation curves for the four impacts on FT-1 and the eight impacts on FT-2 revealed that their curves were in good agreement with each other, and that differences between the properties of the last four impacts started to increase, but the overall trend was consistent (Figure 19b). This suggests that for PU-filled steel crashworthy devices, repeated impacts can also be used to determine the crush curve.

### 3.6. Peak Contact Force vs. Impact Number

**Figure 20:**
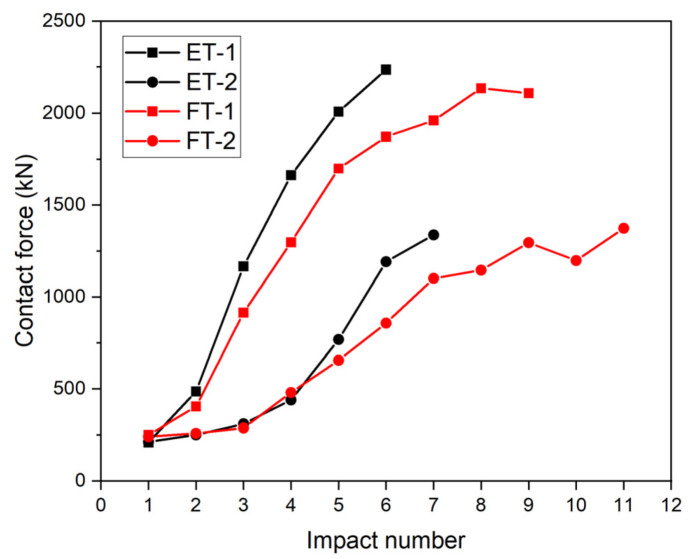
Contact force as a function of impact number.

The variations in the peak contact force for each specimen are plotted in Figure 20. As the number of impacts increased, the peak contact forces on each specimen also increased. In particular, the contact force on specimen ET-1 rose from 209.7 kN after the first impact to a peak of 2237.1 kN. The difference in the contact force was greatest between the first and second impacts and between the second and third impacts. The contact force of the second impact was 2.3 times higher than that of the first impact, and the contact force of the third impact was 2.4 times higher than that of the second impact. The contact force of specimen ET-2 increased from an initial 211.9 kN to a peak of 1337.4 kN, and the maximum difference in the contact force occurred between the fourth and fifth impacts: the contact force of the fifth impact was 1.75 times that of the fourth impact.

The contact force on specimen FT-1 increased from an initial 249.4 kN to a peak of 2135.5 kN, and the greatest difference in the contact force occurred between the second and third impacts: the contact force of the third impact was 2.7 times higher than that of the second impact. The contact force of specimen FT-2 increased from an initial 240.2 kN to a peak of 1374 kN, and the maximum difference in the contact force occurred between the fourth and fifth impacts: the contact force of the fifth impact was 1.67 times that of the fourth impact.

For the ET specimens, the change in the maximum contact force was caused by the change in the structural stiffness as a result of deformation, whereas the change in the maximum contact force for the FT specimens was caused by the steep increase in the stiffness of the filler material as it entered the densification region.

### 3.7. Energy vs. Impact Number

**Figure 21:**
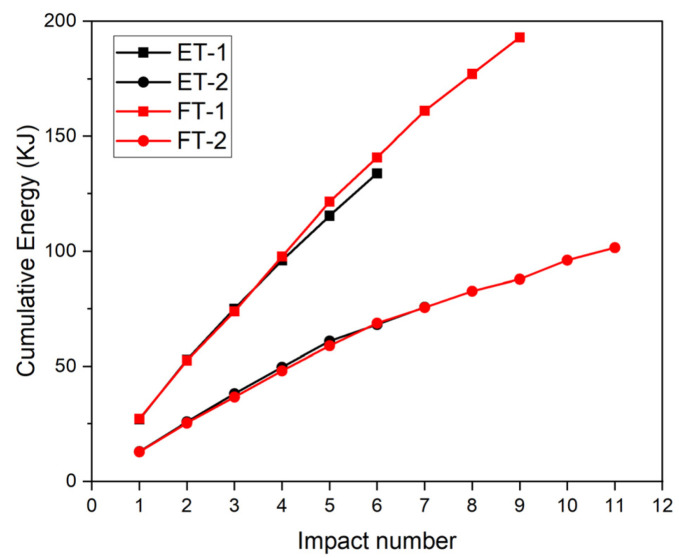
Energy as a function of impact number.

As shown in Figure 21, a comparison between the energy absorption of specimens ET-1 and FT-1 indicated that at the fourth impact, the cumulative energy absorbed by specimen ET-1 was 75 kJ and that absorbed by specimen FT-1 was 73.8 kJ, which were approximately the same values. The accumulated deformation of specimen ET-1 after the fourth impact reached 27.5 cm, the specimen was completely crushed after the fifth and sixth impacts, and the accumulated energy absorption reached 133.8 kJ. However, for specimen FT-1, the cumulative deformation was 25.2 cm for the same amount of energy absorbed (after the fourth impact), which was better than that of specimen ET-1. Owing to the significant increase in stress after the PU was compressed by more than 80%, the cumulative deformation of specimen FT-1 stabilized at 26 cm after nine impacts (seventh to ninth impacts), and the peak contact force stabilized at 1500 kJ, absorbing a cumulative energy of 192 kJ, which is 43.5% higher than that of specimen ET-1.

The energy absorption of specimens ET-2 and FT-2 were also similar. At the seventh impact, the cumulative energy absorbed by specimen ET-2 was 75.6 kJ, and that absorbed by specimen FT-2 was 75.5 kJ. The cumulative deformation of ET-2 at the seventh impact reached 27.4 cm; the specimen was also crushed, and the cumulative energy absorbed no longer increased. However, the cumulative deformation of specimen FT-2 was 25.1 cm, which was significantly greater than that of specimen ET-2. Owing to the significant increase in stress after the PU was compressed by more than 80%, the cumulative deformation of specimen FT-2 stabilized at 25.7 cm after the eleventh impact, the peak contact force stabilized at 1370 kJ, and the cumulative absorbed energy reached 101 kJ, which represents a 33.6% increase relative to that of specimen ET-2.

## 4. Conclusions

Thin-walled tubular structures are widely used in crashworthy devices installed on bridges, and these devices are frequently subjected to repeated impacts at low velocities or low energy. In this study, the dynamic responses of thin-walled and foam-filled tubes subjected to repeated impacts were investigated using the horizontal impact test method. A 12-ton horizontal impactor was used to repeatedly impact the specimens at impact velocities of 2.13 m/s and 1.5 m/s. The test was used to simulate the deformation and force of a crashworthy device structure subjected to repeated impacts by massive ships traveling at low speeds. The contact force on the crashworthy devices was measured directly using piezoelectric sensors, and the speed and deformation were obtained by integrating the contact force–time curves.

The horizontal impactor used in this study was a large-tonnage absolute-stiffness impactor that was used primarily to control the effect of the impactor on the performance of the crashworthy devices. Under actual use conditions, the nonlinear stiffness of the ship leads to a complex coupling with the crashworthy devices, damaging the ship itself, which are different conditions from those of the tests conducted in this study.

The primary conclusions of this study are summarized as follows.

(1) Filling materials are commonly used to enhance the energy-absorbing capabilities of thin-walled structures. According to this study, the filler materials must be crushed in a specific region to support thin-walled structures and increase their ability to absorb energy. The strain must exceed 0.8 for the PU material to be functional.
(2) The experimental evidence showed that the four-phase deformation model is derived from the six-phase deformation model, and that the kinetic energy of the impact primarily influences the deformation modes of thin-walled tubes.
(3) PU materials effectively mitigate the sharp increase in stiffness that results from the collapse of a thin-walled structure, thereby lowering the contact force when the structure is compressed.
(4) Foam-filled tubes can enter a pseudo-shakedown state even after low-speed impacts, which is significant for studying the residual performance of crashworthy devices subjected to repeated impacts.

For future research, the following actions are proposed:

(1) Study the dynamic response of tubes with different wall thicknesses under the same filled material.
(2) Low-density PU is used in this study, which can be used to study the dynamic response of PU with different densities and mechanical properties under repeated impacts.
(3) Study the difference in the dynamic responses of thin-walled structures of hexagonal and octagonal shape, as well as circular structure.
(4) Study the applicability and accuracy of the crush curves of thin-walled and filled structures obtained by using multiple impact tests.
(5) Study the characteristics of energy absorption in thin-walled and filled structures under large velocity impacts (4–5 m/s).

## Figures and Tables

**Table 1:** Impact test parameters for each specimen.

Specimen	Filled	Impact Mass m_s_ (kg)	Impact Velocity v_s_ (m/s)	Impact Kinetic Energy E_o_ (kJ)
ET-1	No	12,000	2.13	27.2
ET-2	No	12,000	1.5	13.5
FT-1	Yes	12,000	2.13	27.2
FT-2	Yes	12,000	1.5	13.5

**Table 2:** Experimental results for specimen ET-1.

No.	Impact Mass m_s_ (kg)	Impact Velocity v_s_ (m/s)	MD (mm)	PD (mm)
1st	12,000	2.121	20.9	18.0
2nd	12,000	2.140	10.9	7.7
3rd	12,000	2.132	3.8	1.5
4th	12,000	2.131	3.0	0.3
5th	12,000	2.134	2.6	0.1
6th	12,000	2.151	2.8	0.1

**Table 3:** Experimental results for specimen ET-2.

No.	Impact Mass m_s_ (kg)	Impact Velocity v_s_ (m/s)	MD (mm)	PD (mm)
1st	12,000	1.520	11.6	10.2
2nd	12,000	1.521	9.2	6.9
3rd	12,000	1.520	7.1	5.5
4th	12,000	1.522	5.1	2.9
5th	12,000	1.531	3.4	1.1
6th	12,000	1.535	2.2	0.5
7th	12,000	1.516	1.8	0.3

**Table 4:** Experimental results for specimen FT-1.

No.	Impact Mass m_s_ (kg)	Impact Velocity v_s_ (m/s)	MD (mm)	PD (mm)
1st	12,000	2.122	18.4	15.2
2nd	12,000	2.131	10.4	6.4
3rd	12,000	2.132	7.1	2.5
4th	12,000	2.118	3.5	1.1
5th	12,000	2.121	3.2	0.3
6th	12,000	2.123	2.7	0.1
7th	12,000	2.124	3.2	0.4
8th	12,000	2.118	3.0	0.0
9th	12,000	2.131	3.1	0.0

**Table 5:** Experimental results for specimen FT-2.

No.	Impact Mass m_s_ (kg)	Impact Velocity v_s_ (m/s)	MD (mm)	PD (mm)
1st	12,000	1.509	10.6	9.2
2nd	12,000	1.523	8.6	7.0
3rd	12,000	1.519	6.9	4.2
4th	12,000	1.516	5.1	2.3
5th	12,000	1.525	4.0	1.1
6th	12,000	1.520	3.0	0.9
7th	12,000	1.520	2.6	0.4
8th	12,000	1.525	2.3	0.2
9th	12,000	1.522	2.4	0.1
10th	12,000	1.514	2.6	0.2
11th	12,000	1.519	2.4	0.0

## Data Availability

The data are confidential due to the project policy. However, it will be made available on reader request.
